# Apatinib has anti-tumor effects and induces autophagy in lung cancer cells with high expression of VEGFR-2

**DOI:** 10.22038/ijbms.2024.74820.16246

**Published:** 2024

**Authors:** Mingtao Liu, Hui Li

**Affiliations:** 1 Department of Pulmonary Medicine, Binzhou People’s Hospital, Binzhou, Shandong, China

**Keywords:** Apatinib mesylate, Apoptosis, Autophagy, Cell proliferation, Lung neoplasms, VEGFR2

## Abstract

**Objective(s):**

This study investigated the inhibitory effect of apatinib on lung cancer cells with high expression of vascular endothelial growth factor-2 (VEGFR-2) and on inducing cellular autophagy and drug resistance.

**Materials and Methods:**

The expression of VEGFR-2 was detected using western blotting and RT-PCR. Cell proliferation was measured using the CCK8 and colony formation assays. The cell apoptosis rate was determined using flow cytometry and tunnel assay. Cellular autophagy was detected by measuring the expression of LC3-II using Western blotting and cellular immunofluorescence. The inhibitory effect of apatinib on lung cancer cells and transplanted tumors was observed after treatment with the autophagy inhibitor chloroquine.

**Results:**

Apatinib dose-dependently inhibited the proliferation of H1975 and H446 cells; it induced apoptosis via the PARP and caspase-3 pathways in H1975 and H446 cells and effectively inhibited the growth of transplanted tumors. Apatinib induced autophagy in a dose-dependent manner in H1975 and H446 cells. The inhibitory effect of apatinib on cells and the promotion of apoptosis were significantly enhanced after treatment with chloroquine. Immunohistochemistry showed that combining apatinib with chloroquine could reduce the expression of CD31 and Ki67 and increase the expression of caspase-3.

**Conclusion:**

Apatinib inhibits proliferation and induces apoptosis in H1975 and H1446 lung cancer cells with high VEGFR2 expression and autophagy in H1975 and H446 cells.

## Introduction

Lung cancer is a malignant tumor with the highest incidence rate (1); the five-year survival of patients with lung cancer is low because early symptoms are not obvious, resulting in a high percentage of advanced lung cancer and loss of surgical opportunities (2). For advanced lung cancer, especially driver gene-negative patients with lung cancer, although immunotherapy has prolonged the survival of a few patients to a certain extent, platinum-based combination chemotherapy is still the cornerstone of treatment for most (3, 4). In recent years, the application of anti-angiogenic drugs, especially when combined with chemotherapy, targeting, and immunotherapy, has greatly improved the survival of patients with advanced lung cancer (5).

The growth and metastasis of cancer cells depend on neovascularization; among many neovascularization pathways, the VEGF-A/VEGFR-2 signaling pathway is the most important (6). Tumor cells directly or indirectly promote vascular endothelial cell proliferation and growth by producing various vascular growth factors. Bevacizumab, the first clinically used anti-angiogenic drug, is a recombinant humanized monoclonal antibody targeting VEGF that blocks the signaling pathway of angiogenesis by specifically binding to VEGF and blocking the binding of VEGF to VEGFR (7). Although bevacizumab has significant anti-angiogenic effects, it has a high risk of bleeding, cardiotoxicity, and other risks and cannot be used in patients with squamous cell carcinoma. Moreover, its use may lead to fatal hemoptysis, and the lack of oral dosage forms of the drug makes clinical application inconvenient; therefore, the use of bevacizumab has been limited in clinical practice. Apatinib is a small tyrosine kinase inhibitor that inhibits VEGFR-2 and suppresses neoangiogenesis in tumor tissues by blocking downstream signaling (8). Apatinib is widely used to treat advanced gastric and gastric cardiac cancers (9, 10). Clinical and basic studies have demonstrated that apatinib has a definite therapeutic effect on lung cancer, including non-small and small cell lung cancers, but extends the survival period of patients with advanced lung cancer to a certain extent (11, 12).

We believe there are two reasons for this poor therapeutic effect. 1) Apatinib may be effective in patients with certain characteristics. Owing to the specific binding of apatinib to VEGFR-2, the efficacy of apatinib is related to the level of VEGFR-2 expressed in the cells or tissues (13). 2) Tumors may develop resistance to anti-angiogenic drugs. Tumors can develop resistance to anticancer drugs due to reduced drug intake, enhanced drug efflux through the overexpression of certain transporters, and inefficient drug penetration into solid tumors (14). Autophagy is the degradation and removal of damaged organelles or misfolded proteins via the lysosomal or proteasomal pathways to maintain homeostasis (15). Recent studies have shown that activation of autophagy in tumor cells can induce resistance to chemotherapy and targeted drugs (16). Regarding autophagy, antiangiogenic drug resistance research has mainly concentrated on bevacizumab (17), whereas research on apatinib drug resistance induced through autophagy has concentrated primarily on thyroid (18), colon (19), and osteosarcoma cancers (20); apatinib-induced autophagy has not been reported to induce resistance in lung cancer cells. 

In this study, two types of cells were selected, representing non-small and small-cell lung cancers; both cell types highly expressed the VEGFR2 protein. We examined the inhibitory effect of apatinib on their proliferation, its mechanism, and whether apatinib could induce autophagy in lung cancer cells with high VEGFR2 expression and their resistance to apatinib.

## Materials and Methos

***Materials***

The cell lines involved in the experiments were purchased from the National Collection of Authenticated Cell Cultures. All cell lines were cultured in RPMI-1640 medium supplemented with 10% fetal bovine serum (Gibco, USA) at 37 ℃ in 5% CO_2_. All cells were passaged for less than three months before renewal from frozen early passage stocks obtained from the indicated sources. Apatinib was obtained from Selleck (s2221, China), dissolved in DMSO, and diluted in RPMI 1640 medium for *in vitro* studies. Apatinib mesylate was obtained from Hengrui Medicine Co., Ltd. (China) and used for animal testing. Immunoprecipitation assay buffer (Applygen, Beijing, China) containing PMSF (Solarbio) was used for western blot analysis. The following primary antibodies were used to detect protein expression levels: primary antibodies against VEGFR-2 (CTS, 2472); LC3B (Abcam, ab48394); PARP (Abcam, ab74290); caspase- 3 (Abcam, ab2302); β-actin (Abcam, ab8227); CD31 (Abcam, ab28364); Ki67 (CTS, 9449); and cleaved caspase-3 (CTS, 9664). The secondary antibodies were purchased from CST. The CCK-8 kit was purchased from Biyuntian (China). Total RNA was extracted using the TRIzol reagent (Invitrogen, USA). Reverse Transcription Reagent Kits (Takara) and SYBR Green Mix (Takara) were used for RT-PCR analysis. The Annexin V-FITC-PI apoptosis detection kit (BD, USA) and TUNEL (Yeasen, China) were used to determine apoptosis using flow cytometry and tunneling, respectively. TX-100 (Sigma) was used for immunofluorescence. The autophagy inhibitor, chloroquine, was purchased from Selleck Chemicals.

***Western blotting***

Western blotting was used to detect VEGFR-2, the autophagy-related protein LC3-II, and apoptosis-related proteins in lung cancer cells. RIPA lysate was added to the cells, cell proteins were extracted, and protein concentrations were determined using the BCA kit and polyacrylamide gel electrophoresis, membrane transfer, and closure. The primary antibodies were incubated overnight, followed by a secondary antibody with horseradish peroxidase (HRP) at room temperature for 1 hr. Protein bands were detected via enhanced chemiluminescence, and β-actin was used as an internal control. The band strength was determined using ImageJ software. All tests were repeated three times to ensure the accuracy of the study.

***Quantitative real-time PCR (qPCR) analysis***

Total RNA was isolated using the TRIzol reagent (Invitrogen, USA). Reverse transcription was performed using a Reverse Transcription Reagent Kit (Takara, Japan). Real-time PCR was performed using the SYBR Green mix (Takara, Japan). The expression of VEGFR-2 mRNA was calculated and normalized using the 2-ΔΔCt method relative to GAPDH.

***Cell proliferation assay***

The toxicity of apatinib in different cell types was detected using a CCK-8 assay. Cells (3000) were plated in 96-well plates and cultured at 37 °C in a 5% CO_2_ incubator for 24 hr before treatment. Cells were treated with different concentrations of apatinib for 24–72 hr. CCK-8 reagent was added, and the culture plates with CCK-8 were incubated in a 5% CO_2_ incubator at 37 ℃ for 2 hr, followed by OD detection using a spectrophotometer. The processed data were analyzed, proliferation curves were plotted, and the IC_50_ values of apatinib against different cells were calculated.

***Colony formation assays***

For the colony formation assay, 1000 cells were plated in 6-well plates and cultured at 37 °C for two weeks. During this process, the fluid was changed every three days, and the cell status was observed. One milliliter of crystal violet dye solution was added to each well, and the cells were incubated for 10–20 min, washed several times with PBS, dried, and photographed with a digital camera.

***Assessment of apoptosis***

Cells were exposed to various concentrations (0, 10, and 20 μM) of apatinib for 24 hr. The effects on apoptosis were detected using flow cytometry. The cells were washed two times with PBS, and 4**×**binding buffer was diluted in 1**×**PBS. The 1**×**binding buffer (100 µl) was added to each tube, the cells were resuspended by blowing with a pipette gun, and the dyes were added while avoiding light. Annexin V and PI (5 µl each) were added, and the cells were incubated in the dark at room temperature for 15 min. Then, 1×binding buffer (300 µl) was added and mixed well, and the cells were transferred to a 5 ml flow tube in the dark within 1 hr for online detection. The TUNEL assay kit was used to detect the apoptotic effect of apatinib on the cells, following the manufacturer’s instructions.

***Immunofluorescence assay***

Cells were treated with medium without apatinib and medium containing 10 μM and 20 μM apatinib for 24 hr, then washed three times with PBS, fixed with 4% paraformaldehyde for 15 min, permeabilized with 0.5% Triton X-100 for 20 min at room temperature, and incubated with goat serum for 30 min. LC3B primary antibody was added, and the cells were put in a wet box at 4 ℃ overnight. Diluted fluorescent secondary antibodies were added, and the cells were incubated in a wet box for 1 hr. DAPI was added and incubated for 5 min in the dark. The slices were sealed with a sealing solution containing an anti-fluorescence quencher and then observed under a fluorescence microscope to collect images.

***Tumor xenograft mouse models***

Nude mice were used to establish an animal model. All animal experiments were conducted at the Animal Center of Shandong University. Appropriate anesthetics, painkillers, and euthanasia were used to minimize the pain and discomfort experienced by the animals during the experiment. Different lung cancer cell suspensions were prepared *in vitro* at approximately 5×10^7^/ml. A single-cell suspension (0.2 ml) was inoculated into the right armpit of the nude mice. Routine feeding continued until the grafted tumors increased to 50 to 100 cm^3^. The intervention trial was divided into two independent parts to observe the tumor-suppressive effect of apatinib on the tumor graft of different lung cancer cell constructs and the effect of apatinib combined with the autophagy inhibitor chloroquine. In the first part of the trial, 15 nude mice were randomly divided into three groups: nude mice were administered saline, 80 mg/kg apatinib, or 120 mg/kg apatinib by gavage once daily for 21 consecutive days. In the second part, fifteen nude mice were randomly divided into three groups: nude mice were given saline gavage, 120 mg/kg apatinib gavage, and 120 mg/kg apatinib gavage combined with 60 mg/kg chloroquine intraperitoneal injection for 21 consecutive days. The apatinib dose was selected based on the literature (18). The volume of the transplanted tumors was measured every three days, tumor suppression curves were plotted, and the mice’s body weights were recorded. On the 21st day, the mice were euthanized by cervical dislocation under anesthesia. The tumor tissues were fixed in 4% paraformaldehyde for immunohistochemical analysis.

***Animal-ethic information***

The Animal Ethics Committee of Shandong University approved all animal tests. The mice’s initial body weight ranged from 18 to 20 g. Appropriate anesthetics, painkillers, and euthanasia were used to minimize the animals’ pain and discomfort during the experiment. No mice died during the administration period; the drug’s safety was high.

***Immunohistochemical staining***

The grafted tumor tissues were fixed in 4% paraformaldehyde for 3–4 hr, dehydrated, embedded, and prepared as wax blocks and sections. The wax blocks were deparaffinized at room temperature and repaired with thermal antigen; 3% hydrogen peroxide was added dropwise, goat serum blocking solution was added, and primary antibodies (CD31, Ki67, and caspase-3) were added to cover the tissues and incubated overnight. Secondary antibodies were added drop-wise and incubated at room temperature for 10 min. DAB color development and staining were terminated if appropriate; sections were rinsed with tap water and photographed under a microscope. 

## Results

***Expression of VEGFR2 protein and mRNA in five lung cancer cells***

**Figure 1:**
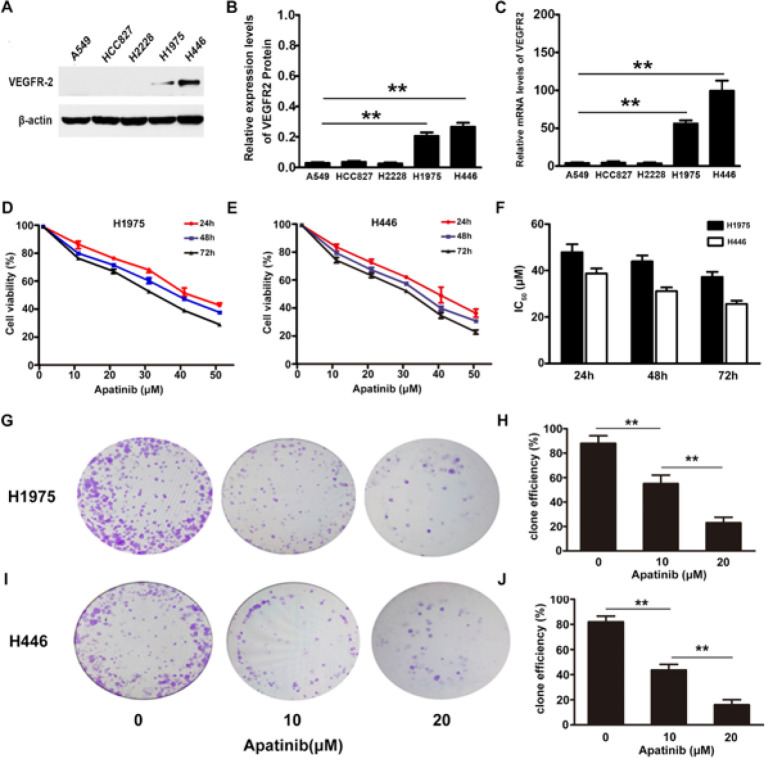
Effects of apatinib on the proliferation of cells with high expression of the VEGFR-2

The VEGFR2 protein is highly expressed in H1975 and H446 cells, with a relative expression content of 0.20 ± 0.08 and 0.28 ± 0.05, respectively. Among the five lung cancer cell lines selected for this study, VEGFR2 expression was highest in these two (Figures 1A and B). The qPCR results showed that in both cell types, the mRNA content for VEGFR2 was high (Figure 1C).

***Inhibitory effect of apatinib on proliferation***

The inhibition of the two lung cancer cell lines increased with increasing apatinib concentration and over time (Figures 1D and E). The IC_50 _values of apatinib for H1975 and H446 increased over 24 hr to 47.9 ± 3.4 μM and 38.7 ± 3.2 μM, respectively. As time progressed, the IC_50_ value of apatinib decreased (Figure 1F). We performed colony-forming experiments to further observe the effect of apatinib at different concentrations on the proliferation of the two lung cancer cell lines. The colony-forming experiments showed that the proliferation of these two cell types could be effectively inhibited by apatinib. In the 0, 10, and 20 μM apatinib concentration groups, the number of cell colony formations was (H1975: 88 ± 6.2, 55 ± 7.0, 23 ± 4.6; H446: 82 ± 4.6, 44 ± 4.5, 16 ± 4.0) (Figures 1G–J).

***Effect of apatinib on apoptosis***

**Figure 2:**
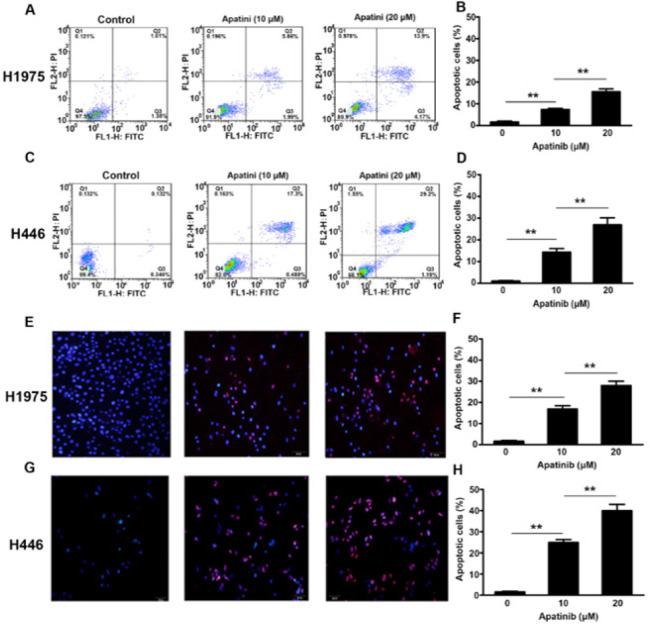
Effects of apatinib on the apoptosis rates of H1975 and H446 lung cancer cell lines were detected using flow cytometry and a TUNEL assay

The flow cytometry result showed that the apoptotic rates of the two cell types in 0, 10, and 20 μM groups were (H1975: 1.63 ± 0.71%, 7.34 ± 0.97%, 15.57 ± 2.21%; H446: 1.02 ± 0.41%, 14.3 ± 2.90%, 26.93 ± 5.56%). The apoptosis-promoting effects on both cell types became more pronounced with increasing apatinib concentrations (Figures 2A–D). The results of the TUNEL assay for the apoptosis-promoting effects of apatinib in both cell lines were similar to those of the flow cytometry assay. The apoptotic ratio of these cells in 0, 10, and 20 μM groups was H1975: 1.7 ± 0.62%, 16.9 ± 2.56%; 27.9 ± 3.56; H446: 1.57 ± 0.58%, 24.9 ± 2.29%, 39.97 ± 5.17% (Figures 2E–H).

**Figure 3:**
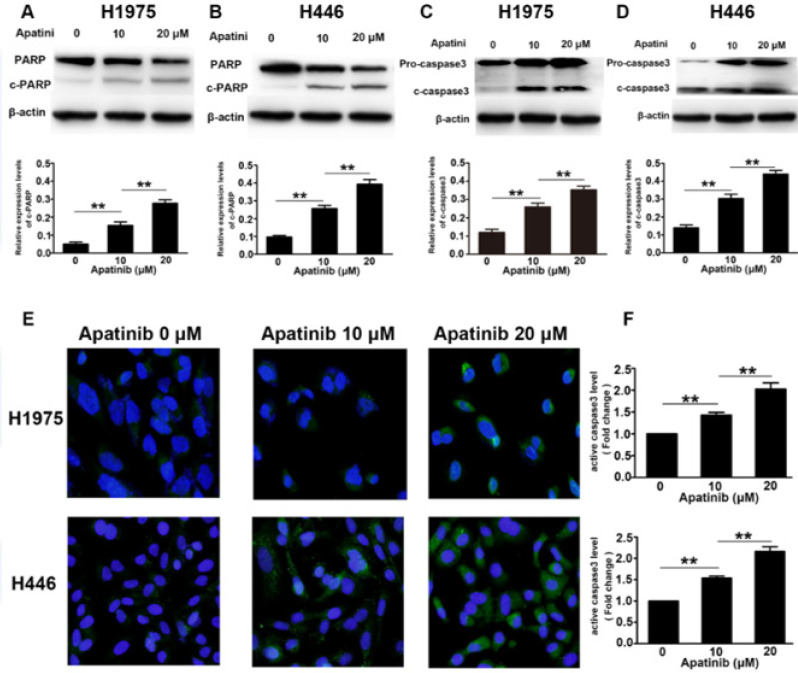
Effects of apatinib on the expression of apoptosis-related proteins were tested using western blotting and immunofluorescence

Western blot and immunofluorescence were used to detect the expression of apoptosis-related proteins (caspase-3 and c-PARP). The results showed that as the concentration of apatinib increased, the expression of the two apoptotic proteins significantly increased (Figures 3A–D). Immunofluorescence results showed that as the concentration of apatinib increased, the cells’ caspase-3 fluorescence expression intensity increased significantly. We further confirmed that apatinib induced apoptosis by activating the caspase-3 pathway in a concentration-dependent manner (Figures 3E and F).

***Inhibitory effect of apatinib on transplanted tumor***

**Figure 4:**
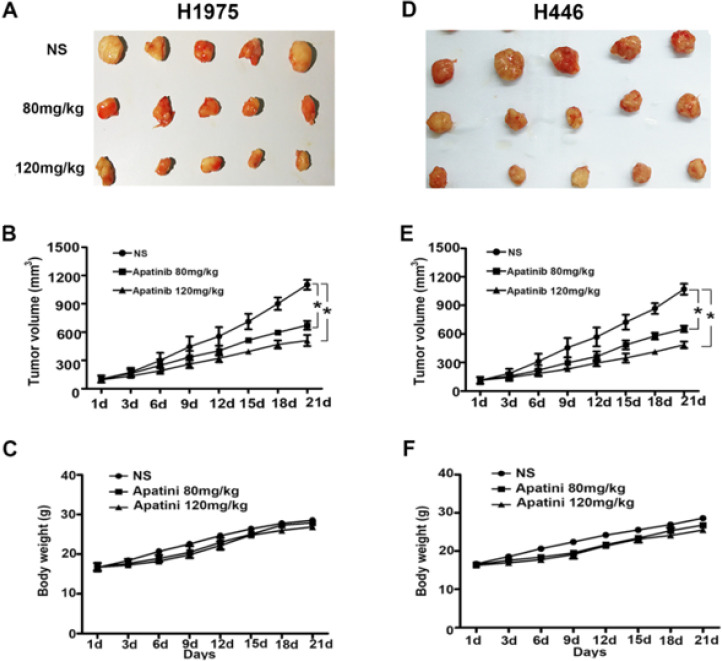
Apatinib inhibits tumor growth in H1975 and H446 xenograft models. BALB/c nude mice xenografted with H1975 and H446 lung cancer cell lines were treated with vehicle or apatinib (80, 120 mg/kg, po, qd) for 21 days

Compared with that in the control group, the 80 mg/kg and 120 mg/kg apatinib gavage treatment groups had significantly inhibited growth rate of transplanted tumors. After three weeks of treatment, the tumor volumes of the control, 80 mg/kg, and 120 mg/kg groups were (1101.8 ± 52.3 mm3, 672.2 ± 46.9 mm3, 510.5 ± 59.1 mm3) for H1975, while that of H446 cells was (1070.2 ± 56.5 mm3, 653.0 ± 32.8 mm3, 485.5 ± 34.9 mm3) (*P*<0.05) (Figures 4A, B, D, and E). The weight of the nude mice in each group was higher than before the intervention. On the 21st day after the intervention, there was no difference in the body weight of nude mice in each group (*P*>0.05) (Figures 4C and F).

***Autophagy induced by apatinib***

**Figure 5:**
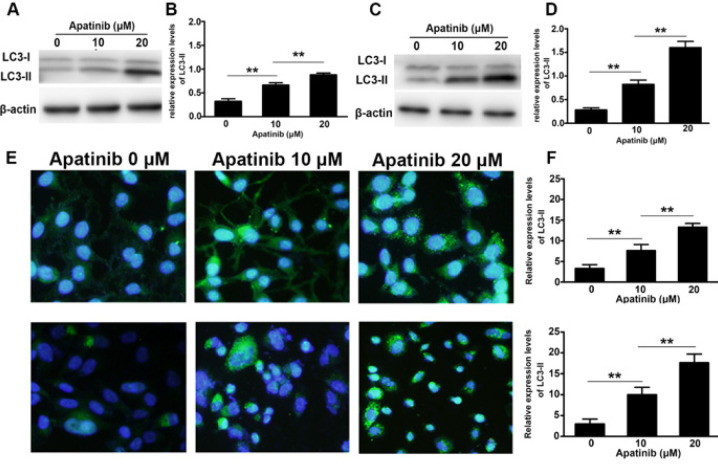
Autophagy induced by apatinib in H1975 and H446 cells

Autophagy was induced in H1975 and H446 cells by apatinib at certain concentrations. With an increase in concentration, autophagy expression became more obvious. Western blotting showed that the expression of LC3-II protein in the two types of cells was significantly increased in both cell types. The relative expression of LC3-II protein in the control group, apatinib 10, and 20 μM groups was (H1975: 0.47 ± 0.03, 0.72 ± 0.02, 0.89 ± 0.05; H446: 0.38 ± 0.02, 0.83 ± 0.05, 1.71 ± 0.06). Compared to the control, the apatinib intervention showed a statistically significant difference; the expression in the high-dose group was more significant (*P*<0.01) (Figures 5A–D). Similar results were obtained with cell immunofluorescence. The proportion of cells with LC3-II expression of more than five points after apatinib intervention was significantly higher than that in the control group, and the effect of apatinib was more significant in the high-dose group, with a statistically significant difference (*P*<0.01) (Figures 5E and F).

***Changes in sensitivity of apatinib to H1975 and H446 cells after the combination of autophagy inhibitors***

**Figure 6:**
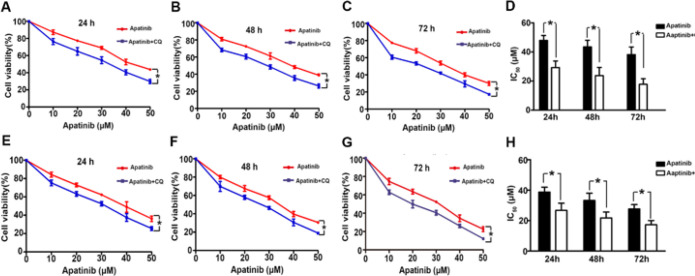
Inhibition by apatinib of H1975 and H446 cells when combined with autophagy inhibitor chloroquine

Apatinib combined with the autophagy inhibitor chloroquine (CQ) significantly increased the inhibition of apatinib in both cell types; the IC_50_ values of apatinib in both cell types were significantly reduced. The IC_50_ values of apatinib combined with CQ were (H1975: 29.5 ± 4.38 μM, 23.85 ± 2.94 μM; 17.8 ± 1.88 μM; H446: 26.7 ± 4.7 μM, 21.7 ± 3.9 μM, 17.3 ± 2.7 μM) at 24, 48, and 72 hr. The IC_50_ value of apatinib combined with CQ was significantly lower than that of apatinib monotherapy (*P*<0.05) (Figure 6). 

**Figure 7:**
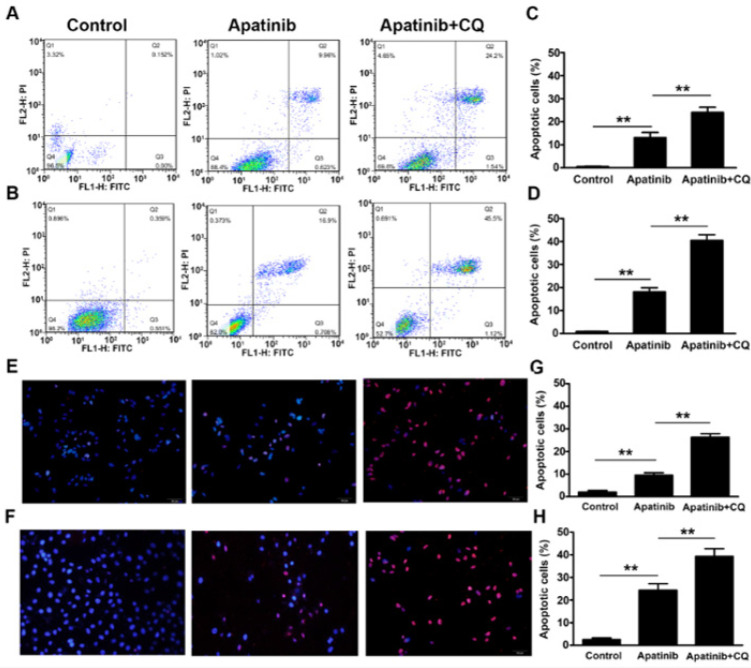
Effect of 10 μM apatinib combined with CQ (10 μM) on the apoptosis of the two lung cancer cell types

When combined with CQ (10 μM), the effect of 10 μM apatinib on inducing apoptosis of the two lung cancer cells was enhanced. The results of flow cytometry showed that the apoptotic ratio of the two cell types before and after apatinib combined with CQ was H1975: 13.1 ± 3.78%, 24.0 ± 3.89; H446: 8.17 ± 2.96%, 40.50 ± 4.32, respectively, more significant than apatinib monotherapy (*P*<0.01) (Figures 7A–D). The results of the TUNEL assay were similar to the flow cytometry data. When combined with CQ, the apoptotic ratio of cells increased significantly (*P*<0.01) (Figures 7E–H). 

**Figure 8:**
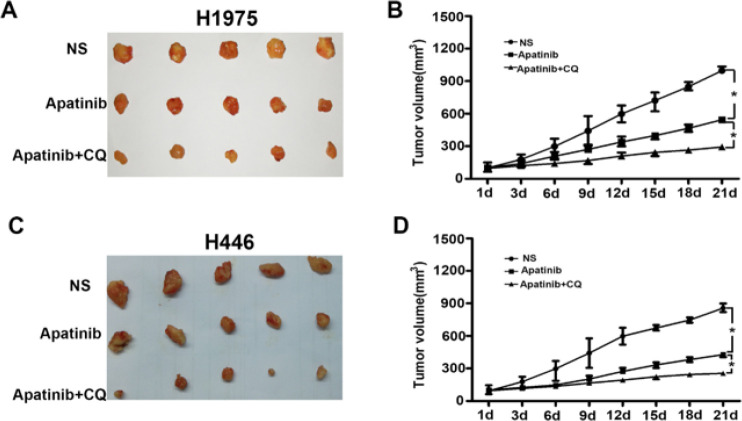
The inhibitory effect of apatinib on H1975 and H446 transplanted tumors in nude mice when combined with CQ

**Figure 9:**
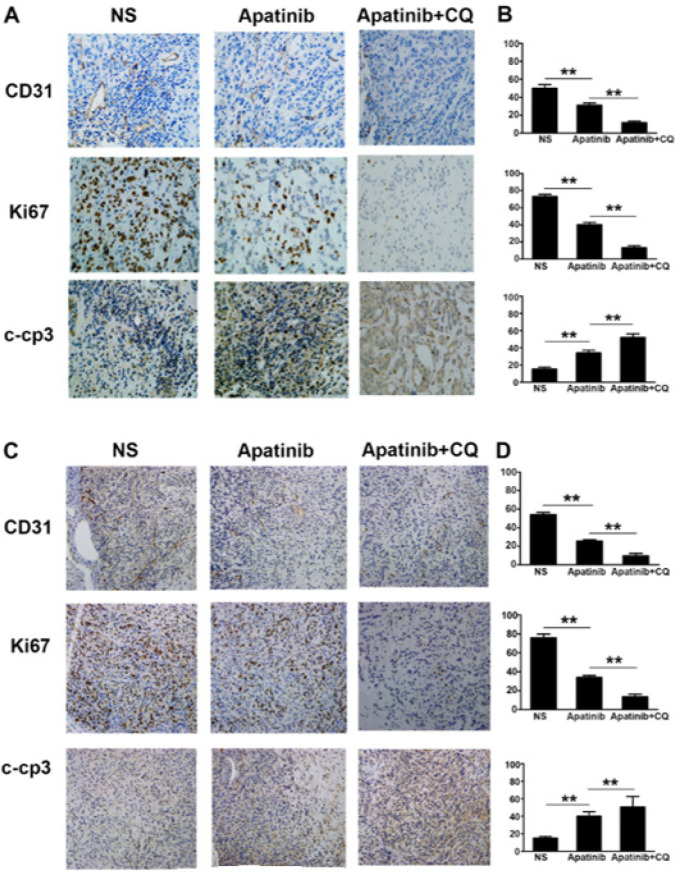
Immunohistochemical staining of the CD31, Ki67, and caspase-3 expression for different groups of xenografted tumor tissues

When combined with CQ, tumor growth inhibition by apatinib on H1975 and H446 transplanted tumors in nude mice was significantly enhanced and different from apatinib monotherapy (*P*<0.05) (Figure 8). The results of immunohistochemistry showed that the number of CD31-positive micro-vessels in each of the five visual fields in the apatinib monotherapy and the combined CQ groups was significantly less than that in the control group; the number of CD31 positive micro-vessels in the combined drug group was significantly lower than that in the other groups (*P*<0.01). Ki67 staining showed that the average number of Ki67-positive cells in each field of vision in the apatinib group was significantly lower than that in the control, especially in the combination group (*P*<0.01). However, caspase-3, which reflects tumor apoptosis, was significantly higher in the apatinib group than that in the control and was more significant in the combined treatment group (*P*<0.01) (Figure 9).

## Discussion

Lung cancer is currently the most prevalent malignant tumor. Patients in the early stages of lung cancer have no obvious symptoms; therefore, most cases cannot be detected early. Most patients diagnosed with lung cancer are already in an advanced stage and have lost the opportunity to undergo surgery. Patients with advanced lung cancer have a short survival time and a poor quality of life. Neovascularization is a significant factor in tumor proliferation, invasion, and metastasis (21). Maintenance of tumor angiogenesis involves various angiogenic factors, among which VEGF is the strongest stimulator (22). VEGF has three types, which need to bind to the corresponding receptor (VEGFR) to exert an important biological role. Three sub-types of VEGFR are observed, among which VEGFR2 is distributed in vascular and lymphatic endothelial cells (23). Moreover, it binds to the corresponding VEGF factor and promotes the formation, migration, and differentiation of new blood vessels and lymphatics. Among these, receptor 2 plays the most significant role (24).

Apatinib is a novel oral small-molecule inhibitor of tyrosine kinases and a specific inhibitor of VEGFR2. Combined with this receptor, it can block the signal transduction of VEGF/VEGFR2 and promote the formation of new blood and lymph vessels to effectively control tumor growth, invasion, and metastasis (25). Because apatinib effectively inhibits tumor neovascularization, it can be used to treat a variety of malignant tumors. Recently, the number of clinical studies on lung cancer treatment has gradually increased (9). Most clinical studies have confirmed that apatinib, alone or combined with other treatments, prolongs patient survival with tolerable adverse effects. However, the effect has been limited, slightly prolonging the overall survival (26).

Apatinib treatment has certain limitations. Apatinib may offer a few advantages to a few patients, but there is much heterogeneity in the efficacy of apatinib in patients with advanced lung cancer. Apatinib is an anti-angiogenesis drug acting via binding to the specific VEGFR2 receptor; the expression of VEGFR2 at the tumor level determines its therapeutic effect. Although there are many new blood vessels in the tumor tissue, vascular endothelial cells express VEGFR2; therefore, the difference in VEGFR2 expression in patients with different lung cancers is mainly due to the difference in VEGFR2 expression on the tumor cell surface (27). Therefore, we selected lung cancer cells with high VEGFR2 expression and examined the inhibitory effect of apatinib. Among the five common lung cancer cell lines, H1975 and H446 cells feature high expression of VEGFR2. We confirmed that apatinib inhibited the proliferation of high-expressing VEGFR2 lung cancer cells and transplanted tumors in nude mice in a concentration-dependent manner. Moreover, in a xenograft tumor model of nude mice constructed with H1975 and H446 cells, apatinib significantly inhibited the growth of xenograft tumors; the higher the concentration, the more significant the inhibitory effect. Apatinib treatment at these concentrations did not significantly decrease the weight of nude mice, indicating that apatinib was safe. The study data confirmed that apatinib can indeed induce apoptosis of lung cancer cells with high expression of VEGFR2 and effectively inhibit the proliferation of lung cancer; thus, patients with lung cancer and high expression of VEGFR2 may benefit from apatinib treatment. H1975 cells represent L858R and T790M mutation-type lung cancer cells, while the first-line treatment in patients with primary lung cancer is three generations of the EGFR-TKI osimertinib; however, osimertinib treatment is expensive, requires repeated treatment after a time, and is faced with the problem of drug resistance (28, 29). Therefore, apatinib may be an effective treatment option for these patients. The next step is to conduct relevant clinical studies to confirm the effectiveness of apatinib treatment in this group of patients. H446 lung cancer cells represent patients with small-cell lung cancer. No other effective treatment exists except first-line EP chemotherapy and local radiotherapy (30). Apatinib may be an effective treatment for small-cell lung cancer.

Although apatinib significantly inhibited proliferation in H1975 and H446 cells with high expression of VEGFR2, any drug may develop resistance at a certain time after treatment. In the current clinical application, at the early stage of the treatment process, apatinib has a significant therapeutic effect, with the tumor volume significantly reduced and even necrosis and cavitation in the short term, indicating that the short-term efficacy is significant. After prolonged treatment, the efficacy gradually declines, the tumor volume is no longer reduced, and even local spread and distant metastasis appear. This suggests that a degree of resistance develops after a period of apatinib application; resistance to all drugs for lung cancer is still a problem that must be faced. Research on the mechanism of anti-vascular drug resistance mainly focuses on the emergence of new angiogenic pathways and the recruitment of bone marrow-derived vascular endothelial progenitor cells (31).

In our study, apatinib induced autophagy to induce drug resistance. Autophagy is the main mechanism of self-energy supply in eukaryotes, which is to maintain the homeostasis, development, and nutrient circulation of cells; as a result, parts of the cytoplasm, organelles, or cell membrane are degraded in the process of lysosome digestion. When nutrients are scarce, autophagy is a self-protective mechanism of cells. In the initial study, the role of autophagy in tumor growth inhibition was generally considered; when autophagy genes are lost, autophagy inhibition can lead to the occurrence of tumors, and with the deepening of the study, researchers observed that as the tumor grows, tumor cells adapt to the lack of oxygen, nutrition environment, or resistance to chemotherapy drugs by strengthening the metabolism of autophagy to sustain themselves and to protect themselves, suggesting that autophagy may play a promoting role in tumor growth. Autophagy plays an antagonistic role in tumor treatment. Several studies have shown that resistance to chemotherapy in lung cancer is associated with autophagy. CIRCU has been observed in cisplatin-resistant lung adenocarcinoma cells. After cisplatin treatment, a large number of autophagosomes were observed in the cytoplasm, and cisplatin-induced cell apoptosis was significantly reduced compared to that in the control group. After an autophagy inhibitor or interfering RNA was used to silence the autophagy gene ATG5, the sensitivity of drug-resistant cell lines to cisplatin returned to normal. Only a few studies are available on anti-angiogenic drug resistance in lung cancer cells. Studies on autophagy-induced tumor resistance to anti-angiogenic drugs have mainly focused on bevacizumab; however, recombinant human endostatin has been reported. Studies have shown that bevacizumab increases autophagy in colon cancer cells, leading to drug resistance. Autophagy inhibitors can improve the sensitivity of colon cancer cells to bevacizumab. Another study confirmed that bevacizumab induces drug resistance by inhibiting the AKT-mTOR pathway and increasing autophagy. In a study on esophageal cancer, it was observed that increased autophagy could lead to drug resistance and decreased sensitivity of endodontic cells, whereas inhibition of autophagy could promote the anti-proliferative effect of endostatin. However, research on apatinib-induced drug resistance in tumor cells mainly focused on malignant tumors, such as thyroid cancer, colon cancer, and osteosarcoma, while research on apatinib-induced autophagy in lung cancer cells leading to drug resistance has not been reported.

Our study observed that a certain concentration of apatinib enhanced autophagy in lung cancer cells with high VEGFR2 expression, and the sensitivity to apatinib decreased. Over time, autophagy became more evident. In addition, the experiment confirmed that after combination with the autophagy inhibitor chloroquine, apatinib had a stronger inhibitory effect on the two cell types. Its IC_50 _value was lower than that of apatinib alone. It could better inhibit the proliferation of lung cancer cells by enhancing the effect of apatinib on inducing apoptosis in lung cancer cells. At the same time, it can be seen in animal experiments that apatinib combined with the autophagy inhibitor chloroquine has a more obvious tumor inhibition effect, significantly inhibiting tumor CD31 and Ki67 expression, which can improve the expression of apoptosis-related protein caspase-3, inhibit proliferation, and induce apoptosis. Therefore, inhibiting autophagy and related signaling pathways may be a new way to treat tumors and reduce drug resistance.

(A and B) The relative expression levels of VEGFR-2 protein. (C) The relative mRNA levels of VEGFR-2 in five cell lines. (D and E) The inhibition rate of apatinib on H1975 and H446 lung cancer cell lines at different times. (F) At different times, the IC_50_ values for apatinib in H1975 and H446 lung cancer cell lines. (G and I) The effect of apatinib on the colony formation of H1975 and H446 cancer cell lines. (H and J) As the concentration of apatinib increased, the number of colonies decreased significantly in H1975 and H446 cells compared with that in the vehicle group (**P<0.01).

(A and C) The apoptosis rates for H1975 and H446 cells treated with 0, 10, or 20 μM apatinib for 24 hr were detected using flow cytometry. (E and G) The apoptosis rates of H1975 and H446 lung cancer cell lines treated with 0, 10, or 20 μM apatinib for 24 hr were detected using a TUNEL assay. (B, D, F, and H) With the gradual increase in apatinib concentration, the apoptotic rates of H1975 and H446 cells were significantly higher than those in the control group (**P<0.01).

(A and B) The relative expression of c-PARP in H1975 and H446 cells treated with 0, 10, or 20 μM apatinib for 24 hr was detected using western blotting. (C and D) The relative expression of caspase 3 in H1975 and H446 cells treated with 0, 10, or 20 μM apatinib for 24 hr was detected using western blotting. The relative expression of c-PARP and caspase 3 in the high-concentration group was higher; there was a significant statistical difference in the three groups (**P<0.01). (E and F) The expression of caspase 3 in H1975 and H446 cells treated with 0, 10, or 20 μM apatinib for 24 hr was analyzed via immunofluorescence detection. The caspase-3 fluorescence expression intensity of H1975 and H446 cells was significantly higher than that of the control group (**P<0.01).

(A and D) The photograph of H1975 and H446 xenografts. (B and E) The plots of tumor growth curves. The 80 mg/kg and 120 mg/kg groups can effectively inhibit the proliferation of H1975 and H446 cells; the inhibition effect of the high-dose group is more significant (*P<0.01). (C and F) The changes in body weight over 21 days. No significant difference was observed between the groups (*P*>0.05).

(A and C) The expression of LC3-II protein in H1975 and H446 cells treated with 0, 10, or 20 μM apatinib was detected using western blotting. (B and D) Compared with the control group, the apatinib intervention group has a significant statistical difference, and the expression of the high-dose group is more significant (**P<0.01). (E) Immunofluorescence detected the expression of the LC3-II protein in H1975 and H446 cells treated with 0, 10, or 20 μM apatinib. (F) The proportion of cells with LC3-II expression greater than five points after apatinib intervention was significantly higher than that in the control group, and apatinib was more significant in the high-dose group (**P<0.01).

(A–C) The inhibition by apatinib of H1975 cells was significantly increased when combined with chloroquine at different times (*P<0.01). (E–G) The inhibition by apatinib of H446 cells was significantly increased when combined with chloroquine at different times (*P<0.01). (D and H) The IC_50_ value of apatinib on both cell types was significantly reduced when combined with chloroquine; the two values were not statistically different (*P<0.01).

(A and C) The flow cytometry of the apoptotic ratio in H1975 cells before and after apatinib was combined with CQ; the ratio was significantly enhanced compared to that in apatinib monotherapy (**P<0.01). (B and D) The flow cytometry of the apoptotic ratio in H446 cells before and after apatinib was combined with CQ; the ratio was significantly enhanced compared to that in apatinib monotherapy (**P<0.01). (E–H) The results of TUNEL were similar to those of flow cytometry. When apatinib was combined with CQ, the apoptotic ratio of the two cell types increased significantly (**P<0.01).

(A and C) The photograph of H1975 and H446 tumors. (B and D) The tumor growth curves are for H1975 and H446. Combined with CQ, the inhibitory effect of apatinib on transplanted tumors in nude mice was significantly enhanced and was significantly different from apatinib monotherapy in H1975 and H446 cell lines (*P<0.01).

(A and B) The expression of CD31, Ki67, and caspase 3 in different groups of H1975 tumor tissues. The number of CD31-positive microvessels and Ki67-positive cells in the combined drug group was significantly lower than that in other groups (**P<0.01); the proportion of caspase-3-positive cells in the combined drug group was significantly higher than that in the other groups (**P<0.01). (C and D) The results were similar to those of H446 cells xenografted on tumor tissues.

## Conclusion

Apatinib effectively inhibits proliferation and promotes apoptosis in lung cancer cells highly expressing VEGFR2. Moreover, it induces autophagy in H1975 and H446 cells. Combined with autophagy inhibitors, apatinib enhanced the inhibitory effect on the proliferation and apoptosis of the two lung cancer cell lines and increased the inhibitory effect of apatinib on xenograft tumors in nude mice.
